# Effects of Various Physical Interventions on Reducing Neuromuscular Fatigue Assessed by Electromyography: A Systematic Review and Meta-Analysis

**DOI:** 10.3389/fbioe.2021.659138

**Published:** 2021-08-23

**Authors:** Xiao Hou, Jingmin Liu, Kaixiang Weng, Lisa Griffin, Laura A. Rice, Yih-Kuen Jan

**Affiliations:** ^1^School of Sport Sciences, Beijing Sport University, Beijing, China; ^2^Department of Sports Science and Physical Education, Tsinghua University, Beijing, China; ^3^Department of Kinesiology and Community Health, University of Illinois at Urbana-Champaign, Champaign, IL, United States; ^4^Department of Kinesiology and Health Education, University of Texas at Austin, Austin, TX, United States

**Keywords:** fatigue, intervention, electromyography, exercise, muscle, rehabilitation, recovery

## Abstract

**Introduction:** Various interventions have been applied to improve recovery from muscle fatigue based on evidence from subjective outcomes, such as perceived fatigue and soreness, which may partly contribute to conflicting results of reducing muscle fatigue. There is a need to assess the effectiveness of various intervention on reducing neuromuscular fatigue assessed by a quantitative outcome, such as electromyography (EMG). The objective of this review and meta-analysis was to evaluate the effectiveness of different interventions and intervention timing for reducing fatigue rates during exercise.

**Methods:** The literature was searched from the earliest record to March 2021. Eighteen studies with a total of 87 data points involving 281 participants and seven types of interventions [i.e., active recovery (AR), compression, cooling, electrical stimulation (ES), light-emitting diode therapy (LEDT), massage, and stretching] were included in this meta-analysis.

**Results:** The results showed that compression (SMD = 0.28; 95% CI = −0.00 to 0.56; *p* = 0.05; *I*^2^ = 58%) and LEDT (SMD = 0.49; 95% CI = 0.11 to 0.88; *p* = 0.01; *I*^2^ = 52%) have a significant recovery effect on reducing muscle fatigue. Additionally, compression, AR, and cooling have a significant effect on reducing muscle fatigue when conducted during exercise, whereas a non-effective trend when applied after exercise.

**Discussion:** This meta-analysis suggests that compression and LEDT have a significant effect on reducing muscle fatigue. The results also suggest that there is a significant effect or an effective trend on reducing muscle fatigue when compression, AR, cooling, and ES are applied during exercise, but not after exercise.

## Introduction

Muscle fatigue is defined as a temporary reduction in maximum muscle force or power induced by exercise (Mackey et al., 2008; Carroll et al., 2017). Muscle fatigue can be caused by various mechanisms, including accumulations of metabolites within the muscle and insufficient motor command in the neuromuscular system (Enoka and Duchateau, 2008). Different types of exercise consisting of various movement tasks result in different fatigue processes for a decrease in muscular force capacity (Enoka and Duchateau, 2008). Severe muscle fatigue can cause a decline in muscular function and joint instability (Rowlands et al., 2001; Paschalis et al., 2007) that affects exercise performance (Cheung et al., 2003; Burt and Twist, 2011). Without an effective intervention, muscle fatigue increases risks of injuries and decreases sports performance (Barnett, 2006). However, there is no consensus in the literature regarding effective management of muscle fatigue. Many studies have been conducted based on subjective outcomes, such as perceived fatigue and soreness (Chaffin, 1973; De Luca, 1984; Guo et al., 2017; Dupuy et al., 2018). Therefore, it is important to investigate the effectiveness of various interventions on reducing muscle fatigue for improving exercise performance and avoiding overuse injuries.

Surface electromyography (EMG) is commonly used to assess muscle fatigue (Filligoi and Felici, 1999). Surface EMG can quantitatively measure myoelectrical activity in real-time noninvasively. Several indexes have been proposed to analyze EMG signals to quantify the degree of muscle fatigue, such as the median frequency (MDF), root mean square (RMS), fractal dimension, and average rectified value (Stulen and De Luca, 1978; De Luca, 1984; Kahl and Hofmann, 2016; Hou et al., 2021). Different methods aim to quantify muscle fatigue caused by physiological changes within the muscle. For example, it has been inferred by a mathematical model that fractal dimension has high sensitivity to detect the synchronization of recruited motor units, which mainly reflects central fatigue (Mesin et al., 2009). The frequency domain indexes of EMG signals, such as MDF or mean frequency (MNF), are associated with the nerve conduction velocity of motor unit action potentials, which mainly reflect peripheral fatigue (Chaffin, 1973; De Luca, 1984). Using various EMG indexes to evaluate the effect of different physical interventions on muscle fatigue can characterize the specific benefits of various interventions and provide insight of underlying physiological mechanism.

A number of physical interventions have been proposed to reduce muscle fatigue and lessen the decline in muscular performance caused by exercise, including massage (Arroyo-Morales et al., 2008), cooling (Anaya-Terroba et al., 2010; Pointon et al., 2011; Luomala et al., 2012; Eguchi et al., 2014; Minett et al., 2014), stretching (Mika et al., 2007; Ghasemi et al., 2013; Padilha et al., 2019), electrical stimulation (ES) (Marqueste et al., 2003), AR (Zarrouk et al., 2011; Akagi et al., 2020), compression (Cavanaugh et al., 2015; Shimokochi et al., 2017), and light-emitting diode therapy (LEDT) (Kelencz et al., 2010; Yang et al., 2012; Toma et al., 2018). These physical interventions induce different physiological mechanisms to alleviate muscle fatigue. Massage has been suggested to reduce muscle tension through activating the Golgi tendon organ reflex and increasing blood flow to remove metabolite wastes (e.g., lactate) (Fleckenstein et al., 2017; Korak et al., 2018). Previous studies have demonstrated that a decline in blood pH is highly correlated with muscle fatigue because H^+^ blocks the binding of calcium to troponin causing excitation–contraction coupling failure (Lindstrom et al., 1970; Komi and Tesch, 1979; Bouissou et al., 1989; Brody et al., 1991). Cooling can help to reduce the local inflammatory response associated with muscle fatigue (Ihsan et al., 2016), observed as a reduction in creatine kinase (CK) (Wozniak et al., 2007), C-reactive protein (CRP) (Pournot et al., 2011), and interleukin-6 (IL-6) (Dupuy et al., 2018). Stretching (Kim et al., 2016), ES (Kim et al., 2016), AR (Yoshida et al., 1996; Fairchild et al., 2003), and compression (Cavanaugh et al., 2015; Shimokochi et al., 2017) can increase intramuscular blood flow, which is helpful for removing metabolic wastes from muscles and supplying oxygen to muscles. This procedure reduces the harmful effect of exercise-induced acidosis and is beneficial for the preservation of neuromuscular function. LEDT, as one of photobiomodulation, can alleviate muscle fatigue through increasing the mitochondrial electron transport rate and ATP synthesis (Kelencz et al., 2010; Yang et al., 2012; Toma et al., 2018).

The time of application of the intervention (after exercise, before exercise, or during exercise) is another important factor that can impact effectiveness of reducing muscle fatigue (Morse et al., 2008; Kay and Blazevich, 2009; Ghasemi et al., 2013). For example, cyclic static stretching after fatigue protocols increases MDF and decreases RMS of muscle contraction (Morse et al., 2008; Kay and Blazevich, 2009; Ghasemi et al., 2013). These changes indicate that stretching after exercise can improve muscle fatigue by increasing firing frequency of active muscle, promoting the recruitment of more motor units to produce the same force output. However, several researchers revealed that static stretching during isometric muscle contraction (Eguchi et al., 2014) or resistance exercise (Padilha et al., 2019) could cause a significant decline in MDF, muscle activation, and force output, which may cause a higher magnitude of muscle fatigue. These inconsistent findings may be explained by the fact that static stretching can prolong the time of the muscles remaining under tension (Souza et al., 2013) and can decrease blood flow (Otsuki et al., 2011), thereby decreasing the delivery of oxygen and nutrients to muscles (Poole et al., 1997; Mcdaniel et al., 2012; Freitas et al., 2017).

There are several meta-analyses comparing the effects of different physical interventions on muscle fatigue (Guo et al., 2017; Dupuy et al., 2018), and most of studies assessed biochemical and pain indexes, including delayed-onset muscle soreness (DOMS), CK, inflammatory indicators, and perceived fatigue (Guo et al., 2017; Dupuy et al., 2018). For example, Dupuy et al. (2018) compared the impacts of different recovery techniques after exercise on DOMS, perceived fatigue, IL-6, CRP, and CK. They found that massage was the most powerful procedure in relieving DOMS, IL-6, and CK and perceived fatigue compared with other interventions (e.g., AR, stretching, compression, ES, immersion, contrast water therapy, cryotherapy/cryostimulation, and hyperbaric therapy/stimulation). In fact, these indicators are more of a presentation of generalized fatigue rather than the local muscle fatigue (Cifrek et al., 2009). However, fatigue caused by exercise is observed as a decline of muscular function, which directly affects performance during exercise (Minett and Duffield, 2014). Peak power output of muscle primarily depends on the optimal sequencing and amount of motor unit activation and recruitment (Ross et al., 2001).

Various interventions have been used to reduce muscle fatigue based on evidence from subjective measures (perceived fatigue and soreness) and systematic responses rather than local muscle responses, which may partly contribute to the conflicting outcomes. There is a need to examine the studies using a quantitative index, such as EMG, to assess the effectiveness of various interventions on reducing muscle fatigue. Therefore, the objective of this study was to compare the effectiveness of different types and timing of physical interventions on reducing exercise-induced muscle fatigue through a meta-analysis of the scientific literature.

## Methods

### Search Strategy and Study Selection

This systematic review and meta-analysis was performed according to the Preferred Reporting Items for Systematic Reviews and Meta-Analyses (PRISMA) guidelines and was registered on the international prospective register of systematic reviews (http://www.crd.york.ac.uk/PROSPERO), registration number: CRD42020211627. No similar systematic review protocol exists.

Relevant studies were searched in PubMed, Web of Science, EBSCO, and Cochrane Library using the following keywords: (“recovery” OR “stretching” OR “active recovery” OR “cooling” OR “cold water immersion” OR “cryotherapy” OR “cold exposure” OR “electrostimulation” OR “TENS” OR “compressive garments” OR “massage” OR “immersion” OR “foam rolling” OR “cupping”) AND “fatigue” AND (“EMG” OR “electromyography” OR “electromyogram”) AND (“post-exercise” OR “after exercise” OR “sports training” OR “exercise”). Two independent observers (XH and JL) screened and identified relevant studies through reviewing all titles, abstracts, and full-text articles from the earliest record to March 2021. Any discrepancies were resolved by discussion or with recourse to a third arbitrator (Y-KJ).

### Inclusion Criteria

Based on the principle related to the terms of population, intervention, comparison/control, outcome, and study design (PICOS), studies included in this review met all of the following criteria: (1) participants were healthy people with no muscle, bone, or neuromuscular diseases; (2) the recovery interventions were limited to physical interventions, such as ES, stretching, or cooling; (3) the interventions applied in control groups were rest without physical interventions or placebo treatments in the same duration as the experimental group; (4) the outcomes of the trial included EMG signals (e.g., RMS, MPF, and MNF); (5) only the trials designed as randomized controlled trials (RCTs), crossover trials, and repeated-measure studies were covered; and (6) the selected articles were peer-reviewed publications written in English.

### Exclusion Criteria

Studies were excluded when they met one or more of the following exclusion criteria: (1) reviews, abstracts, case reports, observational studies, or non-peer-reviewed articles, such as dissertation or conference posters; (2) participants were individuals affected by diseases or injury; (3) the recovery intervention involved drug, ingestion, nutritional supplementation, or any other chemical/pharmacological therapies; (4) there was no fatigue protocol to induce muscle fatigue; (5) the fatigue protocol was not induced by exercise (e.g., ES); and (6) insufficient descriptions of research methodology of the study.

### Quality Assessment

The seven domain biases were evaluated by two independent reviewers using the Cochrane Collaboration tool: (1) random sequence generation (selection bias), (2) allocation concealment (selection bias), (3) blinding of participants and personnel (performance bias), (4) blinding of outcome assessment (detection bias), (5) incomplete outcome data (attrition bias), (6) selective reporting (reporting bias), and (7) other bias (Higgins et al., 2011). An included study was graded as either high, low, or unclear bias. Any disagreements were resolved by discussion or consulting with a third arbitrator (Y-KJ).

### Data Extraction

In accordance with the Physiotherapy Evidence-Based Database scale (Bhogal et al., 2005), two independent reviewers extracted the relevant data from each included study as the following: author(s), publication year, country/region, characteristics of participants [e.g., age, height, body mass, years of strength/endurance training, body mass index (BMI), body fat percentage, fat free mass, maximal oxygen uptake (VO_2_max), and maximal work rate], sample size, fatigue protocol, intervention, and surface EMG parameters.

### Meta-Analysis

The Review Manager software (Review Manager 5.3; The Nordic Cochrane Centre, The Cochrane Collaboration) was used to perform the meta-analysis. Outcomes in meta-analysis included the effect of different physical interventions on several parameters of EMG signals, such as RMS, MPF, and MNF. According to the Cochrane Handbook for Systematic Reviews (Higgins and Green, 2008), either post-intervention values (Mean_post−intervention_ ± SD_post−intervention_) of the outcome or changes from baseline (Mean_ofchanges_ ± SD_ofchanges_) were used to calculate the summary statistic value.

For studies that reported SE, the SD was calculated with following formula: SD = SE × √N (Higgins and Green, 2011; Higgins et al., 2011). If studies only presented data as median and interquartile range (IQR), the mean was equivalent to the median, and the SD was calculated as SD = IQR/1.35 (Wang et al., 2016). If the studies reported the data in terms of CI, the SD was calculated by the formula “√*N* × (*l*_upper_-*l*_lower_)/c, in which *l*_upper_ and *l*_lower_, respectively, represented the upper and lower limits of the CI, and c is a constant depending on the CI and the sample size (Huedo-Medina et al., 2006). If the included articles did not report mean and SD, we contacted the authors to request data. The articles were excluded if there was no reply.

Heterogeneity among studies was assessed by the *I*^2^ index. Low heterogeneity was assumed when *I*^2^ ≤ 25%; moderate heterogeneity when *I*^2^ ≤ 50% and >25%; high heterogeneity when *I*^2^ ≤ 75% and >50%; and very high heterogeneity when *I*^2^ > 75% (Huedo-Medina et al., 2006). A fixed-effect model was estimated when a low or moderate heterogeneity was present and a random-effect model was used if the heterogeneity was high or very high. Because the trials used different scales for EMG signals [e.g., RMS (mV), MDF (Hz), and MPF (Hz)], we performed the standardized mean difference (SMD) to analyze the effects. *p* < 0.05 was considered statistically significant. EMG signals like RMS and fractal dimension show an upward trend with the increasing fatigue, whereas EMG signals, such as MDF and MNF, show the opposite trend. Thus, we used a negative value for the summary statistic value of EMG signals presenting an upward trend with increased fatigue. Subgroups were used to analyze the effectiveness of interventions conducted at different time points (before exercise, during exercise, and after exercise) on EMG parameters. When *I*^2^ > 50% and the included data points exceeded 9, a funnel plot asymmetry was used to assess possible publication bias.

## Results

### Search Result

**Figure 1:**
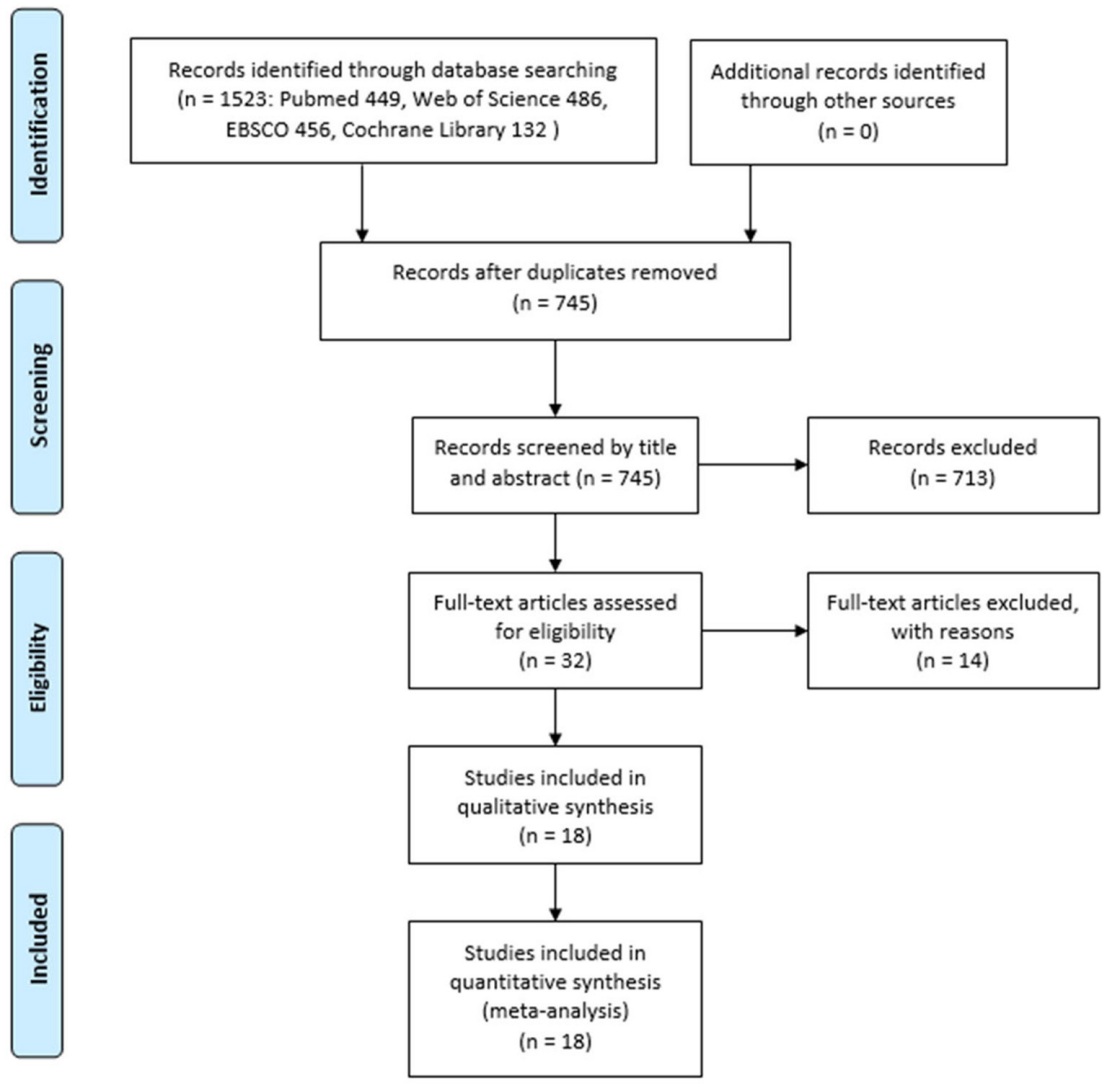
The flowchart of search procedure.

Figure 1 shows the search procedure flowchart. From our initial search of four databases, a total of 1,523 records were identified. Of them, 745 records remained after excluding duplicates. After screening titles and abstracts of those articles, 32 potentially eligible articles remained. After reviewing the full-text articles, 18 articles met the inclusion and exclusion criteria and were pooled in this meta-analysis, and a total of 87 data points (involving 281 participants) were included in this meta-analysis.

### Characteristics of Included Studies

The basic characteristics of the selected articles are summarized in Table 1. The subjects in all studies were young adults aged 17–38 years. The gender of subjects in those 18 studies were: male (*n* = 10, 55.6%), female (*n* = 2, 11.1%), and both genders (*n* = 6, 33.3%). The subjects in seven studies were well-trained adults or athletes, the subjects in eight studies were healthy adults, and the subjects in three studies were physically active adults. In these 18 articles, *most* studies (*n* = 11, 61.1%) evaluated the effect of physical intervention on the fatigue of thigh muscles, two studies (22.2%) assessed the effect of physical intervention on the fatigue of calf muscles, two studies involved thigh and calf muscles simultaneously, two studies involved shoulder or forearm muscle, and only one study involved masseter muscle. Among these 18 articles, four studies reported the effect of cooling on EMG signals of exercise-induced fatigue, three studies involved stretching (static, ballistic, post-isometric relaxation, and traditional), three studies involved AR, three studies used ES, three studies used LEDT, two studies used compression (including compression sleeves, compression garment, and kinesiology tape), and two studies used massage (including icing massage and myofascial release).

**Table 1:** The characteristic of included articles.

**No**.	**References**	**Country**	**Subjects' characteristic**	**Sample size**	**Muscle location**	**Fatigue protocol**	**Type of exercise fatigue**	**Intervention**	**EMG extracted muscle action**	**Outcome**	**Intervention time point**
1	Akagi et al. (2020)	Japan	12 healthy young men (age: 22 ± 1 years, height: 170.0 ± 3.9 cm, body mass: 64.7 ± 13.3 kg)	AR: 12 CON: 12	Gastrocnemius medialis, gastrocnemius lateralis, soleus, tibialis anterior	Two sets of 40 MVCs of hip plantar flexion for 3 s in prone position with a 3-s interval between contractions (i.e., total 80 contractions) with a 1-min interval between sets	Strength training	AR: 10%MVC of hip plantar flexors in the prone position with the hip, knee, and ankle at 0° (0.75 s/time, 30 times/set, six set, 6-s interval between sets, total duration = 5 min) CON: remained in the same position and same duration as AR	Hip plantar flexion MVC	(1). Normalized RMS (MVC%) (2). Normalized MPF (MVC%)	After fatigue protocol
2	Padilha et al. (2019)	Brazil	12 resistance-trained men (age: 29.0 ± 6.1 years, body mass: 84.4 ± 10.3 kg, height: 1.78 ± 0.06 m) with at least one year of strength training experience (5.4 ± 5.1 years of experience, 4.0 ± 0.1 d/wk)	ST: 12 TRAST: 12 CON: 12	Quadriceps (rectus femoris muscle)	Seven sets of 10 isokinetic knee extensions at 60°/s	Strength training	ST: performed 25 s of ST between sets during 40 s rest interval. TRAST: passively rested between sets for 120 s. CON: passively rested between sets for 40 s.	Each concentric knee extension muscle action	(1). Normalized RMS (MVC%) (2). Normalized MDF (MVC%)	During fatigue protocol
3	Toma et al. (2018)	Brazil	18 female participants (age: 25 years ± 2.77, body mass: 58.26 ± 6.57 kg, height: 1.61± 0.05 m, BMI: 22.23 ± 1.59 kg/m^2^)	LEDT: 18 CON: 18	Rectus femoris	A set of 60 concentric contractions of the quadriceps muscle in the full range of standardized range of motion (70°) at 180°/s	Strength training	LEDT: the multi-diode probe was applied to three standardized sites over the rectus femoris muscle of the dominant leg for 3 min CON: placebo treatments for 3 min	60 contractions in fatigue protocol	EFI (EFI =. (sum MDF of last contractions /sum MDF of prior contractions) * 100)	Before fatigue protocol
4	Shimokochi et al. (2017)	Japan	17 healthy male college students (height: 170.7 ± 5.8 cm, body mass: 68.3 ± 9.1 kg, age: 21.8 ± 1.8 years)	CG: 17 CON: 17	Vastus lateralis, vastus medialis, rectus femoris	10 sets of 10 repetitions of maximal isokinetic eccentric and concentric knee extensor contractions, with 30-s rest intervals between sets; with a joint motion range of 45°-90° knee flexion; angular velocity was set 90°/s and 60°/s for knee extensor concentric and eccentric muscle contractions	Strength training	CG: sleep with compression garment for a night after fatigue protocol CON: sleep without compression garment for a night after fatigue protocol (Go to bed at 11:00 p.m. and wake up at 7:00 a.m.)	Knee extensor MVC	(1). RMS (2). MPF	After fatigue protocol
5	Cavanaugh et al. (2015)	Canada	12 university participants (5 females and 7 males)	CS: 12 KT: 12 CON: 12	Vastus lateralis; vastus medialis; biceps femoris	Four sets of unilateral (dominant leg) Bulgarian squats to failure with 1-min rest between sets	Strength training	CS: a Cramer ESS knee CS was worn on the dominant limb. Small, medium, or large CS was worn dependent on the leg volume to ensure a snug fit that would not impinge blood flow. KT: the KT was applied with three vertical strips over the dominant limb vastus lateralis, vastus medialis, and rectus femoris from the base of the patella, encapsulating the patella and then extending to the mid-thigh. The tape was stretched to an additional 50% of its resting length and applied to the skin by an athletic therapist. CON: no garments or tape was worn.	Drop jump from a 50-cm platform	RMS	During fatigue protocol
6	Eguchi et al. (2014)	Japan	16 healthy male undergraduate students (age: 22.8 ± 1.8 years, height: 170.5 ± 6.7 cm, body mass: 64.6 ± 7.9 kg, BMI: 22.2 ± 2.5 kg/m2, BFP: 17.0 ± 4.3, FFM: 53.3 ± 4.8 kg)	SS: 16 BS: 16 CD: 16 CON: 16	Deltoid muscle	kept right arm horizontal, while grasping the dumbbell for 30 s to perform MVC for four sets with 70-s interval between sets	Strength training	SS: static stretching for 30 s between sets BS: ballistic stretching for 30 s between sets CD: transitory icing for 30 s between sets CON: rest between sets	Shoulder abduction isometric muscle contraction in fatigue protocol	MDF	During fatigue protocol
7	Minett et al. (2014)	Australia	Nine moderate- to well-trained, male team-sport athletes (age: 21 ± 2 years, height: 183.3 ± 7.0 cm, body mass: 78.7 ± 8.1 kg)	CD: 9 CON: 9	Vastus medialis and vastus lateralis (mean)	Completed 2 × 35 min bouts of intermittent-sprint exercise in the heat, interspersed by a 15-min mid-exercisepassive recovery period	HIIT	CD: submersion to the mesosternal in 10.0 ± 0.4°C cold water for 20 min CON: received no cooling and sat passively in 32°C and 42% relative humidity for 20 min	Knee extensor MVC	Normalized RMS (% M-wave amp)	After fatigue protocol
8	Ghasemi et al. (2013)	Iran	Nine female basketball players (age: 24.11 ± 3.55 years, body mass: 63.22 ± 10.23 kg, height: 170.78 ±10, BMI: 21.64 ± 2.4)	ST: 9 CON: 9	Triceps surae (Gastrocnemius lateralis; Gastrocnemius medialis; Soleus)	A sustained maximum isometric contraction of triceps surae (with a pressure cuff for reaching fatigue in a short time), the subjects were asked to maintain the maximum plantar flexion peak torque to exhaustion.	Strength training	ST: passively static stretch at 20° of ankle dorsiflexion and held in this position for 20 s, repeated 4 times with a 10-s interval. CON: rest passively for the same duration	Plantar flexion MVC	(1). Pure RMS (2). Pure MDF (3). Normalized RMS (%peak torque) (4). Normalized MDF (%peak torque)	After fatigue protocol
9	Yang et al. (2012)	China	10 healthy right-handed university students (5 males and 5 females, age: 23.7 ± 1.19 years, height: 165.5 ± 6.70 cm, body mass: 55.6 ± 9.38 kg)	LEDT: 5 (4 males and 1 female) CON: 5 (1 male and 4 females)	Right forearm flexors	Performed a sustained fatiguing isometric contraction with the combination of four fingertips except thumb at 30% of MVC until exhaustion.	Strength training	LEDT: Irradiation with LEDT (100 LEDs with wavelength 630nm, spot size 2.5cm^2^, power density 0.048W/cm^2^, and energy density 57.6J/cm^2^) was performed in non-contact mode with the probe held stationary at a vertical distance of 60mm between light source and skin surface for 20 min CON: rest passively for 20 min	Perform the same fatigue task again as that did in fatigue protocol	(1). Normalized ARV (MVC%) (2). Normalized FD (MVC%)	After fatigue protocol
10	Luomala et al. (2012)	Finland	Seven trained male cyclists, (age: 32.4 ± 3.0 years, height: 179.0 ± 2.5 cm, body mass: 80.0 ± 1.9 kg, VO_2_max: 56 ± 3 ml/kg/min, maximal work rate: 325 ± 19 W)	CD: 7 CON:7	Gastrocnemius medialis, vastus lateralis	Cycled with consecutive, non-stop, 10-min cycles of nine min at 60% of their VO2max punctuated by a one-minute sprint at 80% VO2max, the cycling protocol was continuously repeated until exhaustion.	Aerobic exercise	CD: performed with the ice-vest, worn after 30 min of cycling CON: without the ice-vest	During the fatigue protocol *two separate time periods were chosen for analysis:* *(1). period 1: 5 cycling revolutions around the last 15-s point in 9-min 60% VO_2_max cycling* *(2). period 2: 5 s in the middle of sprint performance*	(1). EMG (2). MPF	During fatigue protocol
11	Pointon et al. (2011)	Australia	10 resistance trained male athletes (age: 21 ± 1.6 years; height: 182.2 ± 3.6 cm; body mass: 87.3 ± 9.3 kg)	CD: 10 CON: 10	Vastus lateralis; Vastus medialis, biceps femoris	6 × 25 maximal eccentric/eccentric knee contractions (total contractions: concentric = 150, eccentric = 150) at an angular velocity of 60°/s and 120°/s respectively with each set separated by 1-min rest	Strength training	CD: applied ice cuffs covering the entire surface of the exercised leg (quadriceps, knee and calf) and rested in a supine position for a 20-min duration (water temperature = 0.5 °C). CON: rested in a supine position for 20 min with no ice cuff application	Knee extension MVC	Normalized RMS (% M-wave amp)	After fatigue protocol
12	Zarrouk et al. (2011)	France	Eight elite judo players (age: 18.4 ± 1.4 years, height: 180 ± 3 cm, body mass: 77.0 ± 4.2 kg)	AR: 8 ES: 8 CON: 8	Vastus lateralis, vastus medialis, rectus femoris	Five sets of 10 isokinetic extensions (active phase) and flexions (passive phase) at 120°/s at 80% of the peak torque recorded at 120°/s during the pretraining test	Strength training	AR: in the interval between every sets, 3 minutes of repetitive moderate isokinetic contractions for the right-leg knee extensors, Each athlete executed the active phase in approximately 0.6 s (at 180°/s; 30% peak torque previously recorded at 180°/s) and the passive phase in approximately 1.9 s (at 60°/s). ES: conducted electric stimulation of the quadriceps femoris for 3 minutes between every sets CON: the knee remained in a relaxed state at the starting position (90° of flexion) for 3 min between every sets	(1). 5 Maximal knee extensions at 60°/s (2). 5 Maximal knee extensions at 120°/s (3). 5 Maximal knee extensions at 180°/s	Normalized RMS (% the same action in previous test)	During fatigue protocol
13	Kelencz et al. (2010)	Brazil	30 subjects (seven men and 23 women, age: 23 ± 3 years)	LEDT-SD: 10 CON-SD: 10 LEDT-MD: 10 CON-MD: 10 LEDT-LD: 10 LEDT-LD: 10	Masseter muscle	Masseter muscle 60s MVC	Strength training	LEDT-SD: 8.352 J total energy (1.044 J/point) of irradiation CON-SD: LEDT-SD placebo for the same durations MD-LEDT: 16.704 J total energy (2.088 J/point) of irradiation CON-MD: LEDT-MD placebo for the same durations LEDT-LD: 25.056 J total energy (3.132 J/point) of irradiation LEDT-LD: LEDT-LD placebo for the same durations	masseter muscle MVC	Normalized RMS (% MVC)	After fatigue protocol
14	Anaya-Terroba et al. (2010)	Spain	15 recreational athletes (female, eight) from the Health Science School, University of Granada (age: 19 ± 2 years)	ICEMSG: 15 CON: 15	Vastus medialis, vastus lateralis, rectus femoris	Five isokinetic concentric dominant knee extensions contractions at 60°, 120°, 180°, and 240°/s, subjects were instructed to perform maximum effort on each contraction.	Strength training	ICEMSG: ice massage for 15 min CON: detuned ultrasound for 15 min	Knee extensor MVC	RMS	After fatigue protocol
15	Arroyo-Morales et al. (2008)	Spain	62 healthy active individuals (37 men and 25 women, age: 21.1 ± 2.16 years, body mass: 67.5 ± 1.4 kg, height: 174.3 ± 8.8, BMI: 22.3 ± 1.4, BFP: 15.6 ± 5.4)	MSG: 32 CON: 30	Vastus medialis, vastus lateralis, rectus femoris	Three sets of 30-s Wingate tests on an ergometer cycle with 3-min rest interval between sets.	HIIT	MSG: a whole-body massage myofascial release treatment lasting around 40 min using the Barnes & Upledger approach CON: disconnected ultrasound and magnetotherapy equipment was used for the 40min sham treatment	Knee extension MVC	RMS	After fatigue protocol
16	Mika et al. (2007)	the United States	10 healthy male volunteers (age: 24–38 years, height: 174.7 ±4.66 cm, body mass: 77.5 ± 11.16 kg)	AR: 10 ST: 10 CON: 10	Vastus lateralis	Three sets of dynamic leg extension and flexion (at an angle of 20°-110°) at 50% of MVC, with 30 s of rest between sets	Strength training	AR: light pedaling on the cycle ergometer (10 W) at 60 revolutions/min for 5 min ST: the quadriceps femoris were passively stretched to a point of onset of resistance, “soft end filling”, from this position, the subject performed a prolonged, gentle, isometric contraction against resistance (applied by certified physiotherapist) for 5 secs. Then subjects were told to relax, take a deep breath and exhale completely. During exhalation, the muscle was gently stretched, and from this new position, the procedure was repeated within 5 min, in a supine position. CON: lie down in a relaxed position as a passive rest period for 5 min.	Measured EMG at isometric knee extension at 50% MVC to the point of fatigue after each recovery technique *(the first half effort time and the second half effort time)*	RMS (a). the first half effort time of RMS (b). the second half effort time of RMS	After fatigue protocol
17	So et al. (2007)	China	17 self-reported active young adults (10 male, age: 29.0 ± 2.8 years, height: 175.4 ± 6.6 cm, body mass: 70.3 ± 10.5 kg; 7 female, age = 30.7 ± 4.2 years, height: 164.6 ± 7.5 cm, weight: 56.1 ± 7.3 kg)	ES: 17 CON: 17	Vastus medialis, vastus lateralis, rectus femoris	The knee extension/flexion exercise included two sets of 50 repetitions of full range isokinetic movements, at 120°/s, with a 3-min rest between sets	Strength training	ES: 15 min of transcutaneous electrical acupoint stimulation recovery treatment after the isokinetic exercise CON: 15 min of pseudo TEAS recovery treatment after the isokinetic exercise	A 3-s knee extension (MVC) at 45°flexion before and immediately after the test exercise, and at the 5th, 10th and 15th min of the recovery period.	MDF	After fatigue protocol
18	Marqueste et al. (2003)	France	Seven healthy, untrained men (age: 26.1 ± 2.5 years, height: 173.5 ± 4.5 cm body mass: 71.9 ± 6.2 kg)	ES: 7 CON: 7	Flexor digitorum brevis, Rectus femoris	Keep the preset force level constant (60% of control MVC) until exhaustion	Strength training	ES: stimulated muscles. 6-wk period of ES (30 min/day, 5 days/ week, i.e., 30 sessions), The stimulation pattern consisted of a biphasic symmetrical pulse current (10 V, i.e., submaximal) with a ramp modulation of frequency (4–75–4 Hz) and impulse duration (400–100–400 us) CON: nonstimulated muscles. 6-wk period of non-ES	The fatigue protocol before and after 6-week ES	(1). ΔRMS (2). MDF	Long-term intervention between 2 fatigue protocol

*cm, centimeter; kg, kilogram; AR, active recovery; CON, control; MVC, maximum voluntary contraction; s, second; min, minute; RMS, root mean square; MPF, mean frequency; d, day; wk, week; ST, stretching; TRAST, traditional stretching; MDF, median frequency; BMI, body mass index; m^2^, square meter; LEDT, light-emitting diode therapy; EFI, electromyographic fatigue index; CG, compression garment; CS, compression sleeve; KT, kinesiology tape; SS, static stretching; BS, ballistic stretching; CD, cold intervention; HIIT, high intensity interval training; nm, nanometer; cm^2^, centimeter square; W, Watt; J, Joule; ARV, average rectified value; FD, fractal dimension; VO_2max_, maximal oxygen uptake; ml, milliliter; a-EMG, mean EMG amplitudes; ES, electric stimulation; HT, heating therapy; RM, repetition maximum; SD, small-density; MD, median-density; LD, large-density; MSG, massage; BFP, body fat percentage; FFM, fat free mass*.

As outcome assessments of fatigue, 13 studies reported EMG RMS, six studies reported MDF, three studies reported MNF, and one study reported fractal dimension and average rectified value.

Eleven studies performed the intervention after exercise-induced fatigue, six studies performed interventions during the fatigue protocol, and only one performed the intervention before the fatigue protocol. Most studies (*n* = 15, 83.3%) designed the exercise fatigue protocol using strength training (i.e., maximum voluntary contraction, repeated isokinetic muscle contraction, repeated concentric and eccentric contractions, and resistance exercise), two studies (11.1%) utilized high-intensity interval training (HIIT) as the exercise fatigue protocol, and one study (5.5%) used aerobic exercise as the exercise fatigue protocol.

In addition, the quality of included studies was assessed according to the instruction developed by Higgins and Green (2011). The bias in the quality of included studies mainly came from the blinding of participants and personnel (performance bias) and the blinding of outcome assessment (detection bias). There is a major publication bias in cooling, ES, and compression (more than nine data points were included and *I*^2^ > 50%). Thus, the heterogeneity was primarily from the publication bias.

### Effect of Active Recovery on Muscle Fatigue

**Figure 2:**
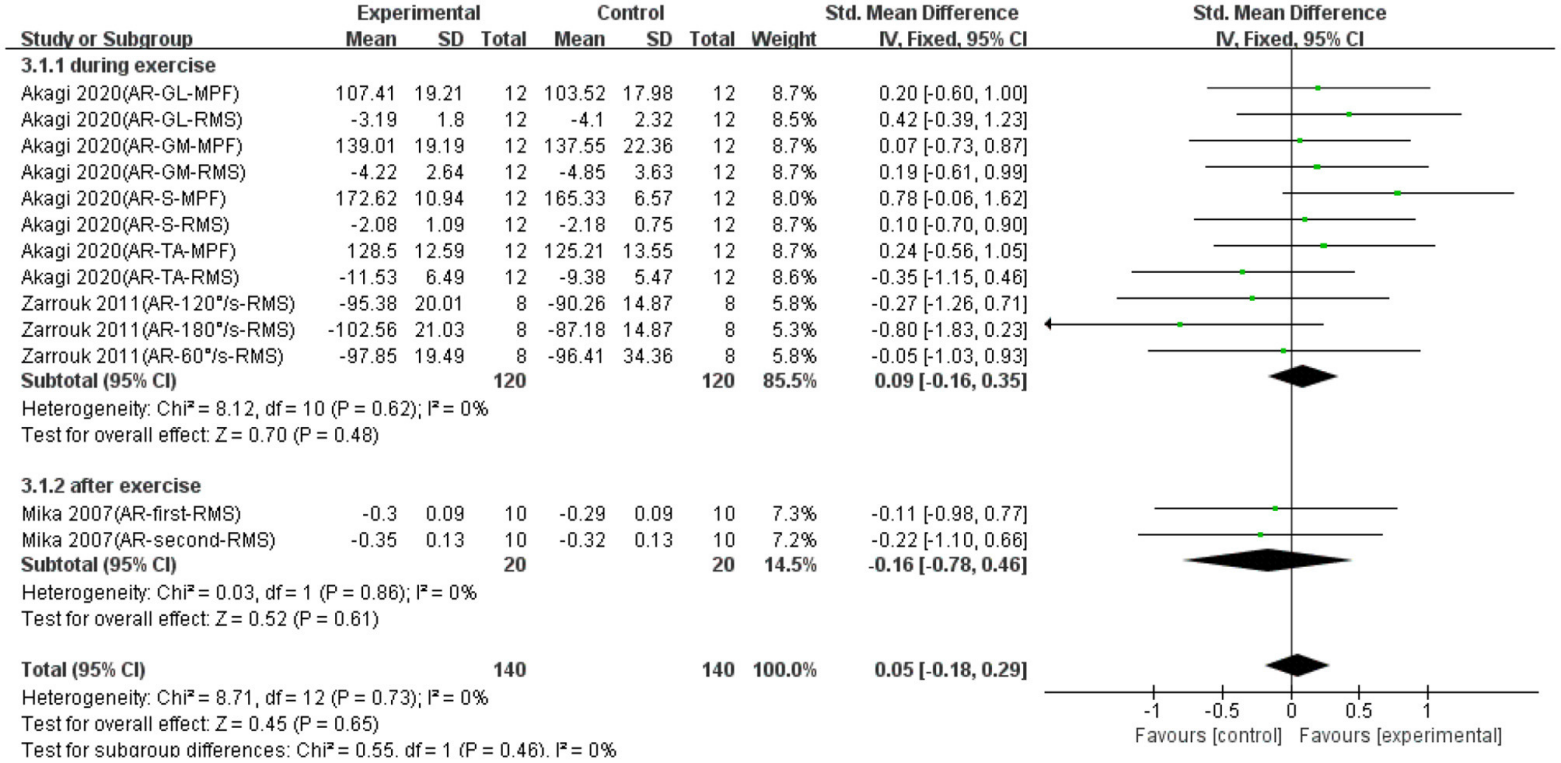
The effectiveness of active recovery (AR) conducted at different time points on electromyography (EMG) signals of exercise-induced muscle fatigue.

Thirteen data points in three studies reported the effect of AR on EMG signals during fatiguing exercise. As shown in Figure 2, no significant difference was found between the AR and control groups based on a fixed model (SMD = 0.05; 95% CI = −0.18 to 0.29; *p* = 0.65; *I*^2^ = 0%). Although not statistically significant, there was an effective trend in the recovery effect of AR on EMG signals of exercise-induced weary muscle. Except that, we made a subgroup analysis based on the conducted time of AR (i.e., during exercise and after exercise). Figure 2 shows that when AR was conducted during exercise, the trend of recovery was effective (SMD = 0.09; 95% CI = −0.16 to 0.35; *p* = 0.48; *I*^2^ = 0%; fixed effect), whereas when AR was conducted after exercise, the trend of recovery was non-effective (SMD = −0.16; 95% CI = −0.78 to 0.46; *p* = 0.61; *I*^2^ = 0%; fixed effect).

### Effect of Stretching on Muscle Fatigue

**Figure 3:**
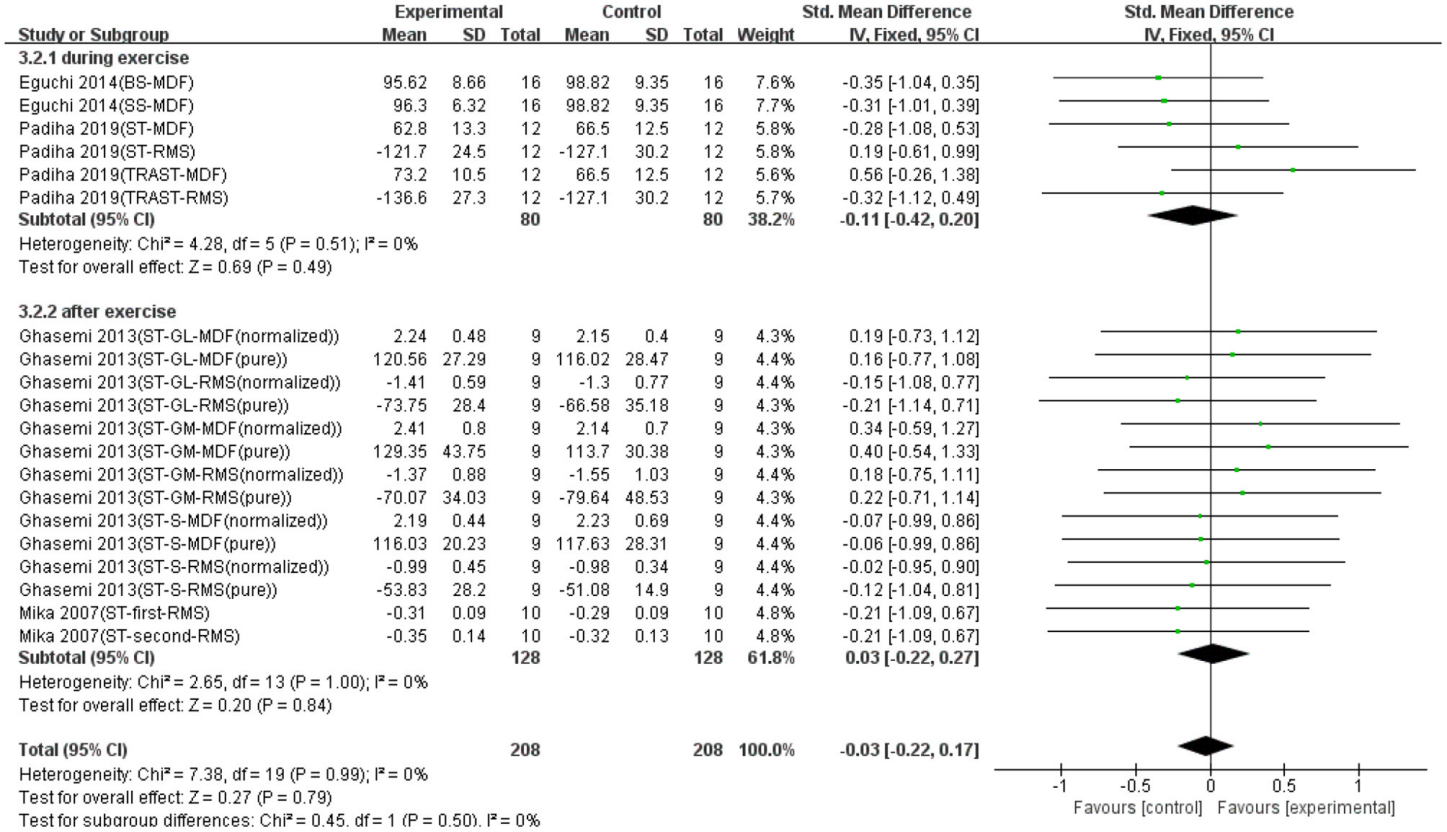
The effectiveness of stretching (ST) conducted at different time points on electromyography (EMG) signals of exercise-induced muscle fatigue.

Twenty data points in four studies demonstrated the effect of stretching on EMG signals of fatigued muscle. Overall, as shown in Figure 3, no significant difference was found between the stretching and control groups based on a fixed model (SMD = −0.03; 95% CI = −0.22 to 0.17; *p* = 0.79; *I*^2^ = 0%). Although not statistically significant, there was a non-effective trend in the recovery effect of stretching on EMG signals of exercise-induced tired muscle. Except that, we made a subgroup analysis based on the conducted time of stretching (i.e., during exercise and after exercise). Figure 3 shows that when stretching was conducted during exercise, the trend of recovery was non-effective (SMD = 0.11; 95% CI = −0.42 to 0.20; *p*= 0.49; *I*^2^ = 0%; fixed effect), whereas when stretching was conducted after exercise, the trend of recovery was slightly effective (SMD = 0.03; 95% CI = −0.22 to 0.27; *p* = 0.84; *I*^2^ = 0%; fixed effect).

### Effect of Cooling on Muscle Fatigue

**Figure 4:**
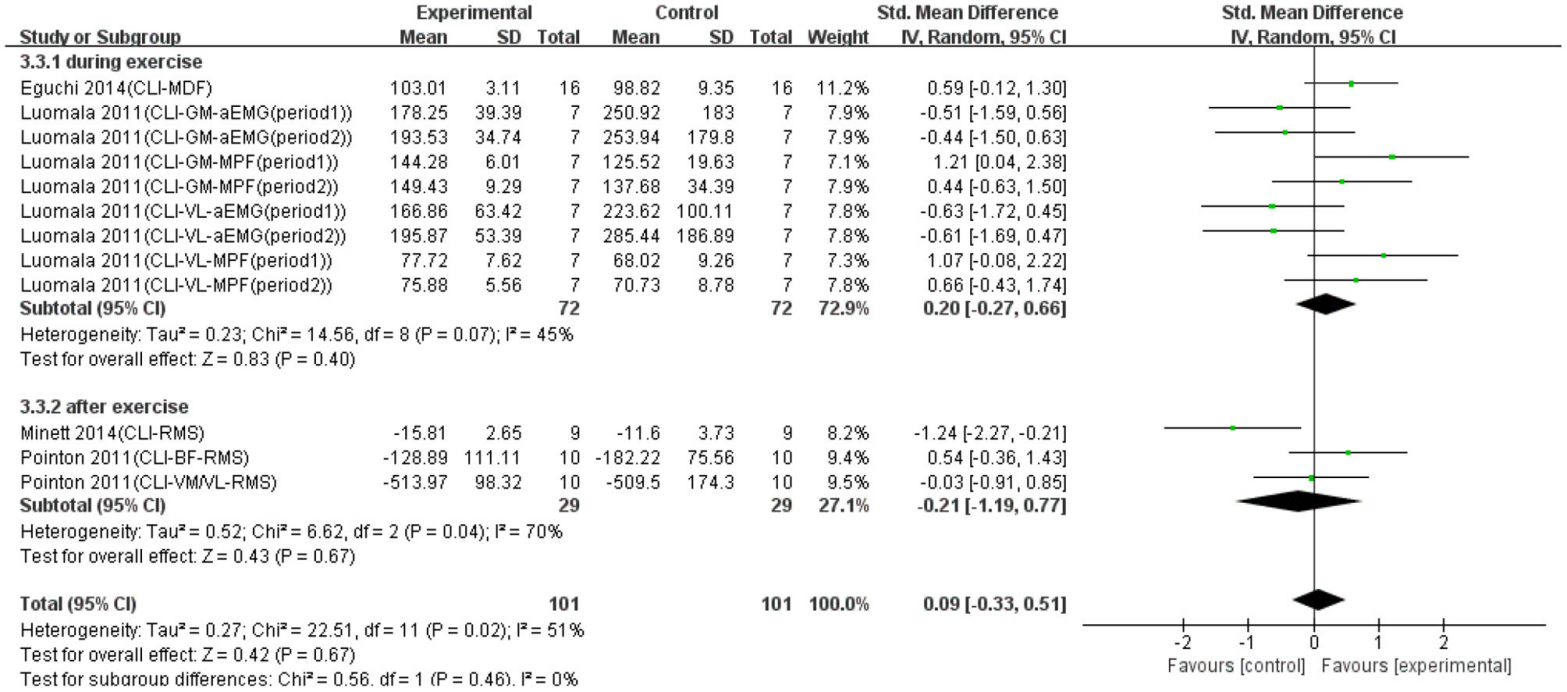
The effectiveness of cooling (CLI) conducted at different time points on electromyography (EMG) signals of exercise-induced muscle fatigue.

Twelve data points from four studies were used to report the effect of cooling on EMG signals of fatigued muscle. Overall, as shown in Figure 4, no significant difference was found between the cooling and control groups based on a random model (SMD = 0.09; 95% CI = −0.03 to 0.51; *p* = 0.67; *I*^2^ = 51%), although not statistically significant, there was an effective trend in the recovery effect of cooling on EMG signals of exercise-induced weary muscle. Except that, we made a subgroup analysis based on the conducted time of cooling (i.e., during exercise and after exercise). Figure 4 shows that when cooling was conducted during exercise, the trend of recovery was effective (SMD = 0.20; 95% CI = −0.27 to 0.66; *p* = 0.40; *I*^2^ = 45%; random effect), whereas when cooling was conducted after exercise, the trend of recovery was non-effective (SMD = −0.21; 95% CI = −1.19 to 0.77; *p* = 0.67; *I*^2^ = 70%; random effect).

### Effect of Electrical Stimulation on Muscle Fatigue

**Figure 5:**
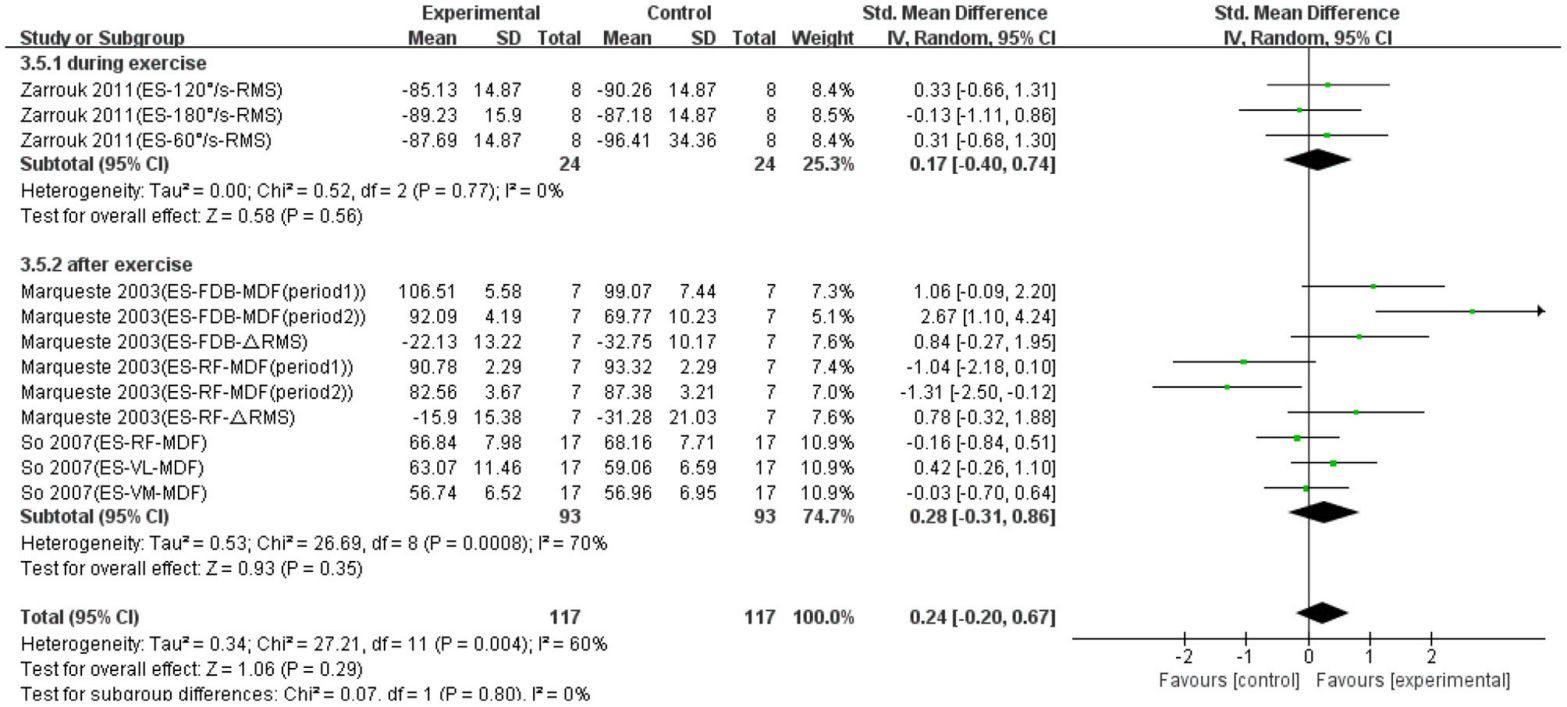
The effectiveness of electrical stimulation (ES) conducted at different time points on electromyography (EMG) signals of exercise-induced muscle fatigue.

Twelve data points from three studies were used to report the effect of ES on EMG signals of fatigued muscle. Overall, as shown in Figure 5, no significant difference was found between the ES and control groups based on a fixed model (SMD = 0.24; 95% CI = −0.20 to 0.67; *p* = 0.29; *I*^2^ = 60%). Although not statistically significant, there was an effective trend in the recovery effect of ES on EMG signals of fatigued muscle. Except that, we made a subgroup analysis based on the conducted time of ES (i.e., during exercise and after exercise). Figure 5 shows that the trend of recovery was effective, when ES was conducted both during exercise (SMD = 0.17; 95% CI = −0.40 to 0.74; *p* = 0.56; *I*^2^ = 0%; random effect) and after exercise (SMD = 0.28; 95% CI = −0.31 to 0.86; *p* = 0.35; *I*^2^ = 70%; random effect).

### Effect of Compression on Muscle Fatigue

**Figure 6:**
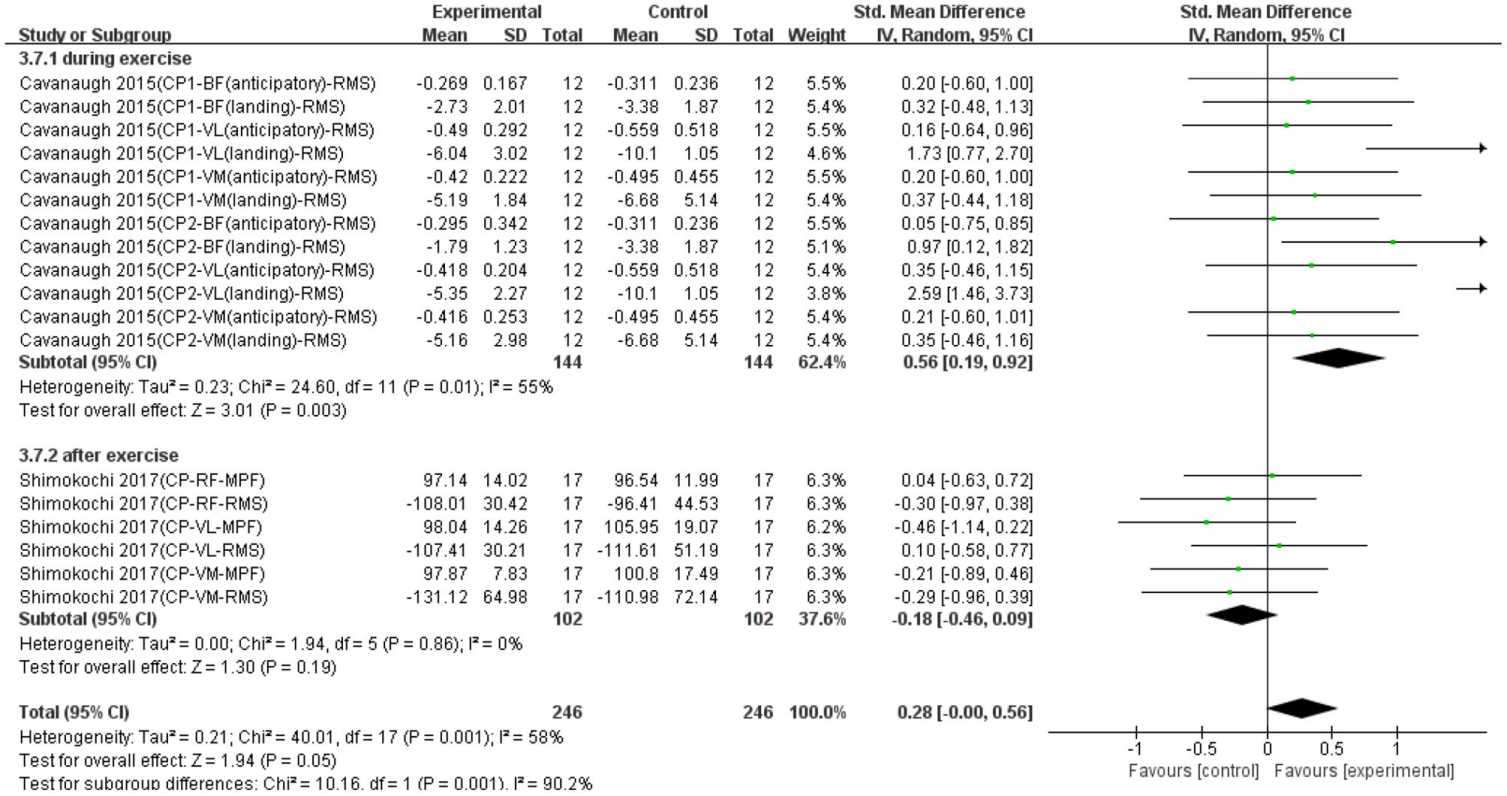
The effectiveness of compression (CP) conducted at different time points on electromyography (EMG) signals of exercise-induced muscle fatigue.

Eighteen data points from two studies were used to report the effect of compression on EMG signals of fatigued muscle. Overall, as shown in Figure 6, there was a significant difference between compression and control groups based on a random model (SMD = 0.28; 95% CI = −0.00 to 0.56; *p* = 0.05; *I*^2^ = 58%), which indicated compression had significantly effective recovery effects on EMG signals of fatigued muscle. Except that, we made a subgroup analysis based on the conducted time of compression (i.e., during exercise and after exercise). Figure 6 shows that when compression was applied during exercise, the recovery effect was significantly more effective (SMD = 0.56; 95% CI = 0.19 to 0.92; *p* = 0.003; *I*^2^ = 55%; random effect). When compression was applied after exercise, the trend of recovery was non-effective (SMD = −0.18; 95% CI = −0.46 to 0.09; *p* = 0.19; *I*^2^ = 0%; random effect).

### Effect of Massage on Muscle Fatigue

**Figure 7:**
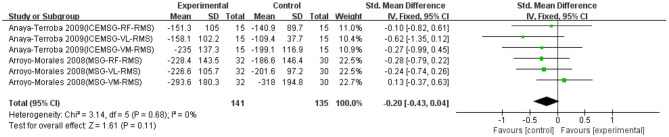
The effectiveness of massage (MSG) on electromyography (EMG) signals of exercise-induced muscle fatigue.

Six data points from two studies were used to report the effect of massage on EMG signals of fatigued muscle. As shown in Figure 7, there was no significant difference between massage and control groups based on a fixed model (SMD = −0.20; 95% CI = −0.43 to 0.04; *p* = 0.11; *I*^2^ = 0%), although not statistically significant, there was a non-effective trend in the recovery effect of massage on EMG signals of fatigued muscle. Because the massage intervention in these two studies were conducted after exercise, the subgroup analysis based on the conducted time was not performed in Figure 7.

### Effect of Light Emitting Diode Therapy on Muscle Fatigue

**Figure 8:**
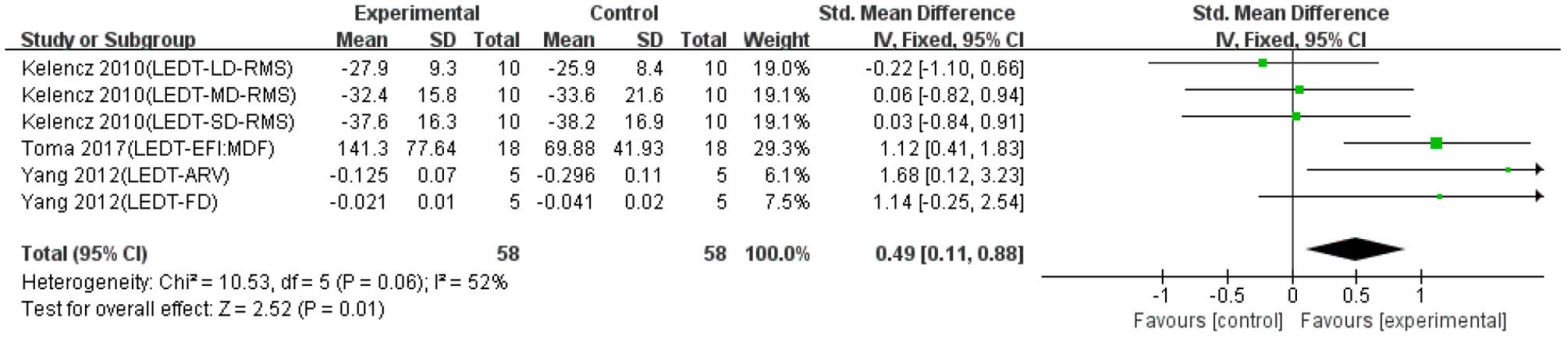
The effectiveness of light-emitting diode therapy (LEDT) on electromyography (EMG) signals of exercise-induced muscle fatigue.

Six data points from three studies were used to report the effect of LEDT on EMG signals of fatigued exercise. As shown in Figure 8, there was a significant difference between LEDT and control groups based on a fixed model (SMD = 0.49; 95% CI = 0.11 to 0.88; *p* = 0.01; *I*^2^ = 52%), which indicated LEDT had significantly effective recovery effects on EMG signals of exercise-induced muscle fatigue. Because only one data point applied LEDT before exercise, the subgroup analysis based on the conducted time was not performed in Figure 8.

## Discussion

In this meta-analysis, data from 18 studies regarding the recovery effects of AR, stretching, cooling, ES, massage, and LEDT on EMG signals of fatigued muscle were, respectively, synthesized. To the best of our knowledge, this is the first study evaluating the effectiveness of various interventions and intervention timing (i.e., before, during, and after exercise) for reducing fatigue as assessed by surface EMG. Among the interventions included in our study, both compression and LEDT seem to alleviate muscle fatigue. The results based on the subgroup analysis of various intervention timing could provide specific evidence for the selection of the optimal time point when applying a certain physical intervention. To be specific, the effectiveness of AR, cooling, and compression during exercise rather than after exercise is positive for reducing muscle fatigue. However, the effectiveness of stretching after exercise rather than during exercise is positive for reducing muscle fatigue. For ES, the effectiveness is positive for reducing muscle fatigue during exercise and after exercise.

The results demonstrated that compression and LEDT had a significant positive effect on the recovery of EMG signals of exercise-induced tired muscle. For compression, research studies have discussed its effect on the recovery of fatigued muscles (Doan et al., 2003; Rider et al., 2014; Cavanaugh et al., 2015), and there were inconsistent results. Most studies did not find the positive impact on relieving muscle fatigue, such as DOMS. However, our review identified the effective influence of compression on the recovery of myoelectrical activity, especially when compression was applied during exercise. It could be explained by the fact that the appropriate pressure produced by compression increases muscle oxygenation (Zajkowski et al., 2002; Agu et al., 2004), and the increased oxygenation could provide more energy and oxygen for muscles during exercise. Compression equipment could decrease unnecessary muscle activation during exercise (Miyamoto et al., 2011), which could slow down muscle fatigue and increase neuromuscular efficiency. It has been suggested that compression equipment may prevent muscle from vibrations during physical activities and reduce the wasted muscle force on keeping stability (Miyamoto et al., 2011). Additionally, the increased H^+^ caused by the accumulation of lactate during exercise could inhibit the excitation–contraction coupling and the occurrence of cross bridge of muscle fibers (Allen et al., 2008a,b). Several studies reported blood circulation and blood lactate clearance were increased after compression (Berry and Mcmurray, 1987; Robinson and Gribble, 2008). In our meta-analysis, compression after exercise has no significant recovery effects on the electrophysiological variable. This result may be because the recovery effect of compression conducted after exercise was caused by the muscle-derived mechanisms, which would not be presented in EMG signals rather than the nerve-derived factors in neuromuscular system (Shimokochi et al., 2017).

Another effective physical intervention identified in this meta-analysis was LEDT. Research studies found that LEDT can delay the appearance of muscle fatigue and inhibit the increase in blood lactate and CK activities (Leal Junior et al., 2009, 2011). As one of the photobiomodulation therapies, the positive effect of LEDT on the muscle recovery may be attributed to the increase in ATP synthesis rate, the improvement of peripheral circulation (Ihsan, 2005), and the minimization of oxidative stress (Fujimaki et al., 2003; Fillipin et al., 2005; Leal Junior et al., 2008). These changes could improve myoelectrical activities of muscle fatigue through modifying the physiological properties at the cellular level (Allen et al., 2008b). Future studies need to investigate the potential mechanism of LEDT on the recovery of electrophysiological parameters of exercise-induced muscle fatigue.

Our results provide scientific evidence that the popular interventions (i.e., massage and stretching) applied in exercise fatigue practice had non-effective trend for reducing exercise-induced muscle fatigue. For massage, several studies (not using EMG measurements) demonstrated the effect of massage on reducing regional muscle fatigue using blood lactate concentrations (Robertson et al., 2004) and elevating spinal motoneuron excitability (Sullivan et al., 1991; Cafarelli and Flint, 1992; Morelli et al., 1999), but some did not demonstrated the effectiveness of massage on muscle performance (Tiidus, 1997). In this meta-analysis, we first revealed the non-effective trend of massage on the EMG signals. This contradictory result may be attributed to the limitation that massage is a manual manipulation, and the dose of massage is difficult to quantify. More studies will be needed to assess the dosage of massage on reducing muscle fatigue assessed by EMG (Kerautret et al., 2020).

Stretching is routinely performed in sports and rehabilitation for prevention of muscle injuries and recovery of muscle fatigue (Rodenburg et al., 1993; Guissard et al., 2001; Schulte et al., 2004). It has been demonstrated that stretching has significant effects on the increased flexibility and decreased muscle stiffness (Decoster et al., 2005; Morse et al., 2008; Kay and Blazevich, 2009). In our review, we found that stretching had a noneffective trend on the recovery of EMG signals of muscle fatigue. This may be because stretching can improve internal mechanical properties of muscles (e.g., muscle stiffness) rather than the activity of neuromuscular system, which is directly related to the muscle force output and sports performance (Decoster et al., 2005; Morse et al., 2008; Kay and Blazevich, 2009; Souza et al., 2013).

It is worth mentioning that the results of this meta-analysis are limited to the effects of various physical interventions on EMG indicators of fatigued muscles. The findings of this study only refer to the effect of an intervention on reducing muscle fatigue assessed by EMG and do not apply to other benefits, such as reduced muscle stiffness (Jan et al., 2021) and neuromuscular efficiency (Milner-Brown et al., 1986; Prieske et al., 2013; Mueller et al., 2017). The physical interventions that have no effects on EMG signals may be useful for another outcome of muscle fatigue. For example, it has been shown that massage can significantly decrease the severity of muscle soreness and produce positive psychological effects, although it has no significant effects on functional performance of muscle (Weerapong et al., 2005). Neuromuscular efficiency is a measure to evaluate the functional state of muscles and is commonly interpreted as fatigue ratio (Milner-Brown et al., 1986; Prieske et al., 2013; Mueller et al., 2017). In this study, neuromuscular efficiency was not discussed because none of included of this index to evaluate the effectiveness of interventions on reducing muscle fatigue. Future studies may consider to use neuromuscular efficiency to evaluate the effectiveness of various intervention on the strength–activation relationship of muscles.

Despite this is the first meta-analysis reporting the effectiveness of different physical interventions on electrophysiological signals of exercise-induced muscle fatigue, the methodology has limitations. First, the publication bias, low-quality study, and language restriction of included studies may have non-objective influence on the effect. Although we tried to control the source of bias in our meta-analysis, we could not completely avoid this limitation. Second, the major objective of this review is to evaluate the effectiveness of different physical interventions on EMG signals of muscle fatigue, although we reported the different characteristic of exercise fatigue protocols (e.g., strength training, HIIT, and aerobic exercise), we could not eliminate the interaction with the effect of a particular recovery strategy. For example, the effect of a certain physical intervention on muscle recovery from fatigue depends on the type of exercise. Third, due to the limitation of the number of included studies, we did not separate immediate effect from long-term effect of physical interventions on EMG parameters. Most studies included in our review discuss the immediate effect of physical interventions, and only one study (Marqueste et al., 2003) involved the long-term effect of intervention on muscle performance in the fatigue protocol. This factor may also cause inflated estimates of the effect. Fourth, we used the predetermined keywords (i.e., AR, compression, cooling, ES, LEDT, massage, and stretching) for this systematic review; other interventions, such as cupping therapy, were not included due to an insufficient number of high-quality studies. The intervention not included in this review may be effective on reducing muscle fatigue. Future studies may use the Medical Subject Headings (MeSH) to perform systematic reviews. Last, the number of studies included in this systematic review is limited (*n* = 18). Thus, the evidence of effectiveness of interventions may need to be reexamined when more studies are available. The purpose of this systematic review is to evaluate available evidence and provide the first evidence for sports and rehabilitation professionals for choosing interventions on reducing muscle fatigue after exercise.

## Conclusions

This review and meta-analysis suggests that compression and LEDT have a significant recovery effect on the EMG signals of exercise-induced muscle fatigue. In addition, AR, cooling, and ES seem to have an effective trend, whereas massage and stretching are not effective on reducing muscle fatigue. The results of subgroup analysis based on timing effect suggest that there is a significant effect or an effective trend on reducing muscle fatigue when compression, AR, cooling, and ES are applied during exercise, but not after exercise. To the best of our knowledge, this is the first systematic review and meta-analysis examining the effects of various physical interventions on reducing muscle fatigue assessed by EMG.

## Data Availability Statement

The original contributions presented in the study are included in the article/supplementary material, further inquiries can be directed to the corresponding author/s.

## Author Contributions

All authors listed have made a substantial, direct and intellectual contribution to the work, and approved it for publication.

## Conflict of Interest

The authors declare that the research was conducted in the absence of any commercial or financial relationships that could be construed as a potential conflict of interest.

## Publisher's Note

All claims expressed in this article are solely those of the authors and do not necessarily represent those of their affiliated organizations, or those of the publisher, the editors and the reviewers. Any product that may be evaluated in this article, or claim that may be made by its manufacturer, is not guaranteed or endorsed by the publisher.
